# Supplementary material for: E2F1 proteolysis via SCF‐cyclin F underlies synthetic lethality between cyclin F loss and Chk1 inhibition
Source: EMBO J. 2019 Aug 19;38(20):e101443. doi: 10.15252/embj.2018101443 (PMC6792013; doi:10.15252/embj.2018101443)
Supplement: Supplementary file 1 — Expanded View Figures PDF [file EMBJ-38-e101443-s001.pdf]

Expanded View Figures

Figure EV1. Cyclin F loss promotes cell death after checkpoint inhibition (related to Fig 1).

A Cell cycle profile of HeLa and *CCNF* K/O detected by FACS using DAPI.
B Cell cycle distribution of untreated HeLa and *CCNF* K/O cells using combined phospho-histone H3 Serine 10 and EdU staining.
C Cell survival of RPE cells transfected with non-targeting siRNA siNC (negative control) or siCyc F after treatment with Chk1i (LY2603618) at indicated concentrations compared to DMSO-treated controls (NT).
D Cell survival measured using resazurin and compared to controls treated with DMSO (expressed as relative proliferation %). Cells were treated with ATR inhibitors at the indicated concentrations.
E Cell survival of U-2-OS cells transfected with non-targeting siRNA siNC (negative control) or siCyc F after treatment with Chk1i (LY2603618) at indicated concentrations compared to DMSO-treated controls (NT).

Data information: Data are presented as mean ± SD, with at least three independent experiments. *P*-values ($^*P < 0.05$, $^{**}P < 0.005$) were calculated by paired and two-tailed *t*-test.

© 2019 The Authors

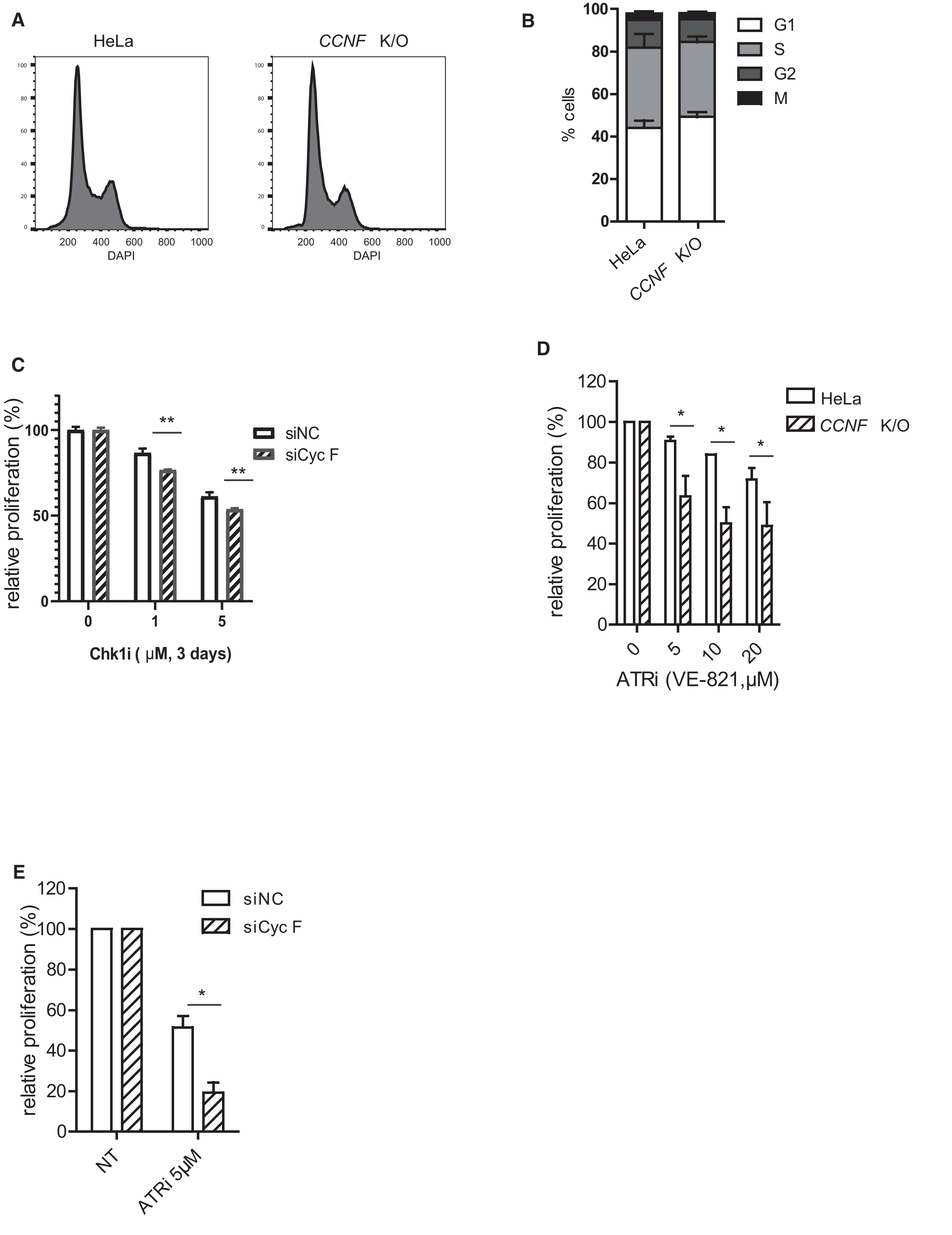

Figure EV1.

 © 2019 The Authors

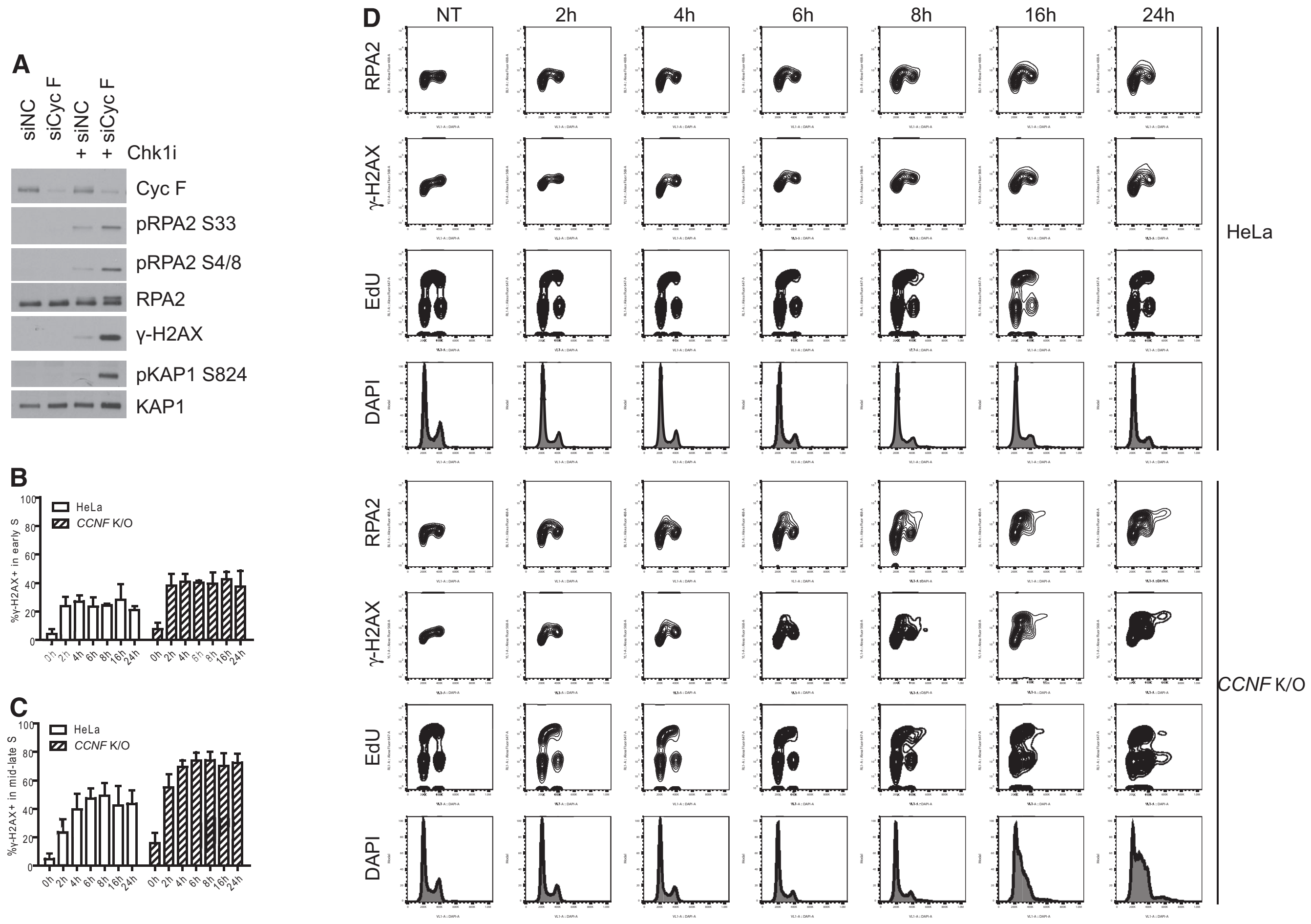

Figure EV2. Loss of cyclin F results in S phase accumulation of DNA damage (related to Figs 2 and 3).

A U-2-OS cells transfected with the indicated siRNA and treated with Chk1i (LY2603618) for 20 h were harvested and lysed using SDS. Indicated proteins were resolved by SDS–PAGE and detected by Western blot (WB).

B Percentage (%) of γ-H2AX-positive cells in early S cells after Chk1i treatment (LY2603618). Early S phase cells were considered having 2n DAPI content and EdU^+. Data from at least three independent experiments were plotted with mean % ± SD.

C Percentage (%) of γ-H2AX-positive cells in mid-late S phase after Chk1i (LY2603618) treatment. Mid-late S phase cells were considered having DAPI 4n staining and EdU^+. Data from at least 3 independent experiments were plotted with mean % ± SD.

D Representative plots of HeLa and *CCNF* K/O cells treated with 1 μM Chk1i for the indicated hours (h). Half an hour before harvesting, cells were incorporated with EdU (10 μM as final concentration). EdU by click reaction, γ-H2Ax and RPA by immunostaining, and DAPI staining were detected by FACS analysis.

© 2019 The Authors

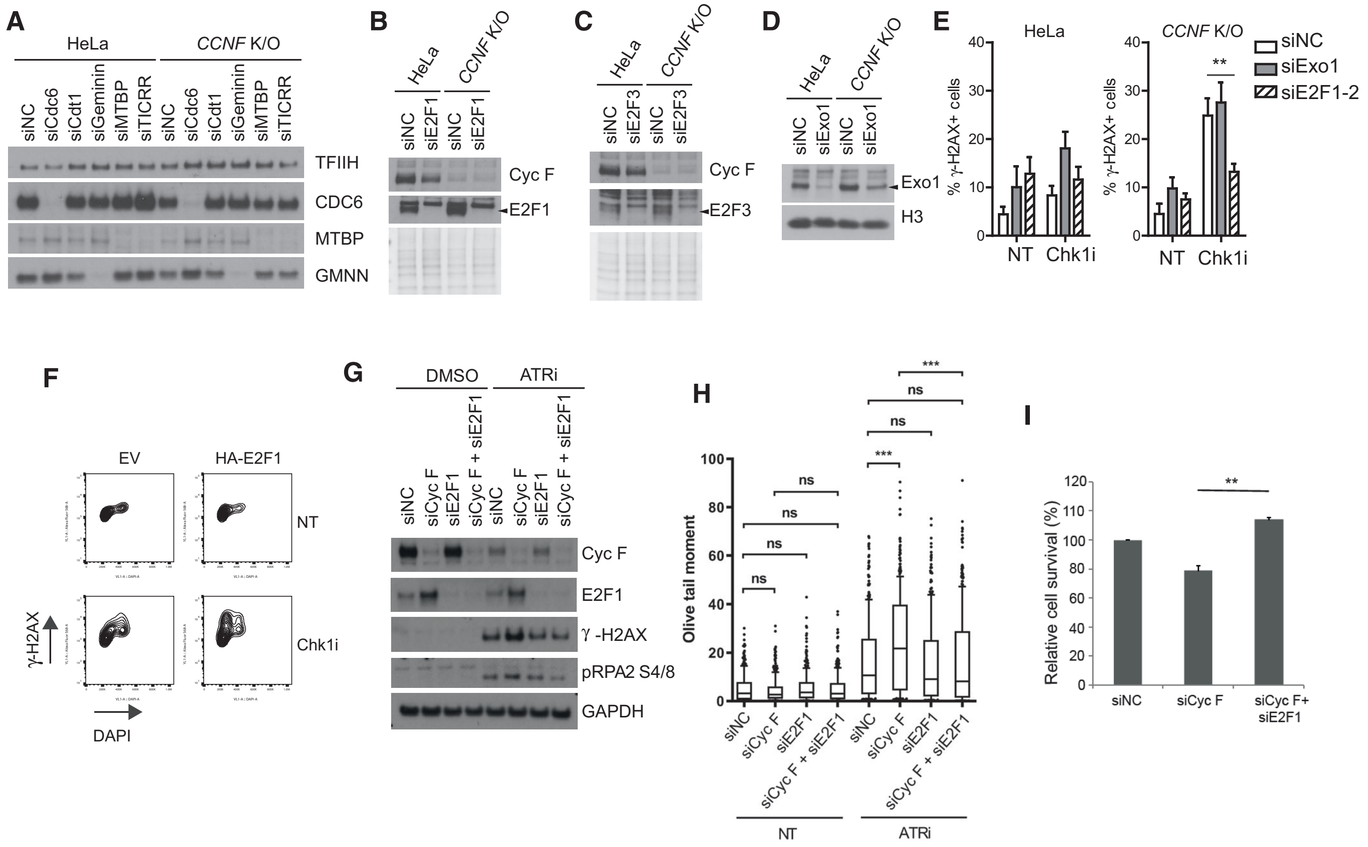

Figure EV3. E2F1 accumulation mediates DNA damage in *CCNF* K/O cells treated with Chk1 and ATR inhibitors (related to Fig 4).

- A HeLa and *CCNF* K/O cells were transfected with the indicated siRNA for 48 h were harvested and lysed using SDS. Indicated proteins were resolved by SDS–PAGE and visualised by WB. The image verifies knockdown efficiency of experiments presented in Fig 4A and B. TFIIH was used as a loading control.
- B WB of indicated proteins as in (A). Ponceau S staining is a loading control.
- C WB of indicated proteins as in (A). Ponceau S staining is a loading control.
- D WB of indicated proteins as in (A). H3 is a loading control.
- E Percentage (%) of γ-H2AX-positive cells in HeLa and *CCNF* K/O after indicated siRNA transfection and 1 μM Chk1i for 20 h. Percent of γ-H2AX-positive cells were plotted as mean % ± SD (**$P < 0.005$). Data were calculated from triplicate experiments using two-tailed paired *t*-test.
- F Representative FACS plots of γ-H2AX versus DAPI signal of HeLa cells transfected with empty vector (EV) or HA-E2F1 untreated or treated with 1 μM Chk1i for 20 h.
- G Cells transfected with non-targeting siRNA (siNC) or indicated siRNAs were left untreated (DMSO) or treated with ATRi (VE-821, 1 μM) for 24 h. Cells were harvested and lysed using a Triton X-100-based lysis buffer. Indicated proteins were resolved by SDS–PAGE and visualised by WB.
- H Quantification of comet tails in alkaline gel. At least 100 cells, across two slides, were analysed in each condition in three biological replicates. Data are shown as medians, with 25/75% percentile range (box) and 10–90% percentile range (whiskers). *P*-values were calculated using the Mann–Whitney test (two-tailed). Olive tail moment = (Tail.mean − Head.mean)*Tail%DNA/100 ***$P < 0.0005$.
- I Cells transfected with indicated siRNAs are subjected to ATR (VE-821, 1 μM) inhibition for 24 h. Percentage survival of each cell line was assessed via resazurin viability assay and plotted as mean % ± SD (**$P < 0.005$). Data were calculated from triplicate experiments using two-tailed paired *t*-test. For simplification, survival rates were normalised to non-targeting control siNC treated with ATRi.

 © 2019 The Authors

Figure EV4. E2F1 is a ubiquitylation substrate of cyclin F at the G2/M transition and after checkpoint inhibition (related to Fig 5).

A A gene set enrichment analysis (GSEA) with enrichment of PID_E2F_PATHWAY, ISHIDA_E2F_TARGETS and HALLMARK_E2F_TARGETS gene signature in HeLa versus *CCNF* K/O.

B HeLa and *CCNF* K/O cells were synchronised by double thymidine block and collected at the indicated hours (h) after release. Indicated proteins were resolved by SDS–PAGE and detected by WB. GAPDH was used as a loading control.

C HEK293T cells were cotransfected with MYC-tagged ubiquitin, and HA-E2F1 in the presence of FLAG-cyclin F WT, FLAG-cyclin F (ΔF) and FLAG-cyclin F M309A as indicated (+). Indicated proteins were resolved by SDS–PAGE and detected by WB. H3 was used as a loading control. Correspond to the input of Fig 5E.

D HeLa and *CCNF* K/O cells were treated with cycloheximide (CHX—50 μg/ml) for the indicated hours (h), harvested and lysed using SDS. Indicated proteins were resolved by SDS–PAGE and detected by WB. TFIIH was used as a loading control.

E HeLa and *CCNF* K/O cells treated with Chk1i for 8 h and MG-132 or MLN4924 for 2 h at the indicated concentrations were harvested and lysed using SDS. Indicated proteins were resolved by SDS–PAGE and detected by WB. H2B was used as a loading control.

© 2019 The Authors

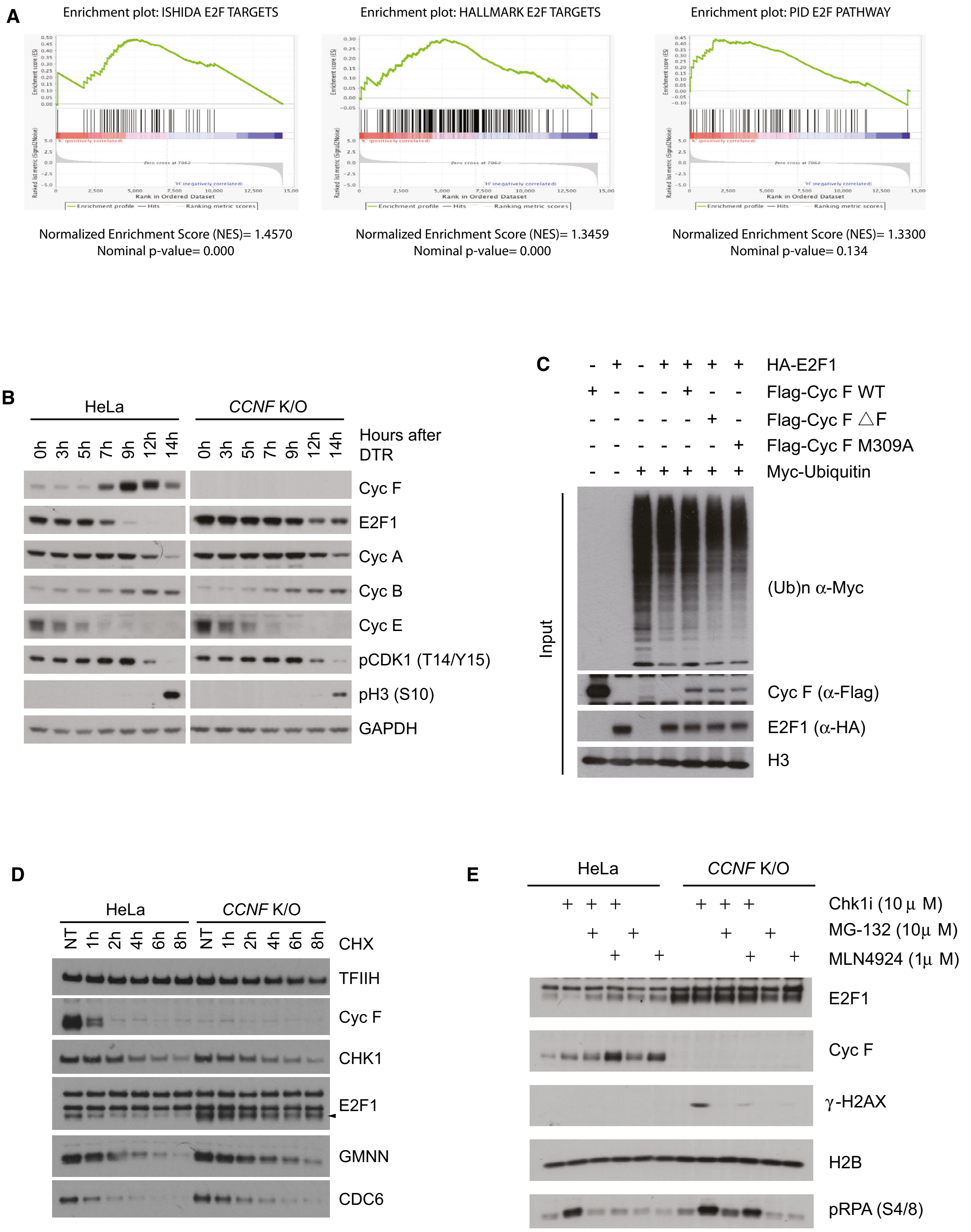

Figure EV4.

 © 2019 The Authors

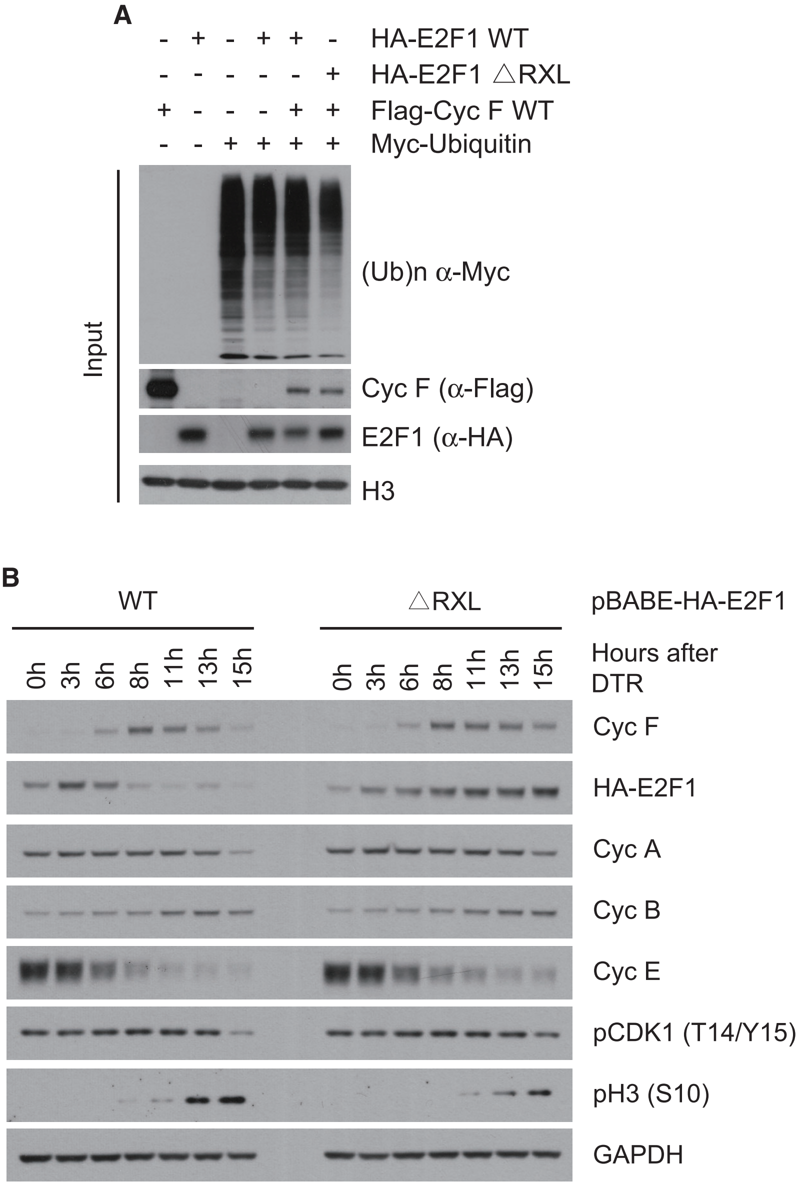

Figure EV5. A mutant of E2F1 lacking a CY motif is not degraded by cyclin F at the G2/M transition (related to Fig 6).

A HEK293T cells were cotransfected with MYC-tagged ubiquitin and HA-E2F1 or HA-E2F1 ΔRxL lacking the CY motif as indicated (+). Indicated proteins were resolved by SDS–PAGE and detected by WB. H3 was used as a loading control. Correspond to the input of Fig 6B.

B HeLa cells stably expressing HA-E2F1 WT and HA-E2F1 ΔRxL under the control of a retroviral promoter were synchronised by double thymidine block and harvested at the indicated hours (h) after release. Indicated proteins were resolved by SDS–PAGE and detected by WB. GAPDH was used as a loading control.

© 2019 The Authors
